# Diagnostic Value of Neutrophil-Lymphocyte Ratio for Predicting the Severity of Acute Pancreatitis: A Meta-Analysis

**DOI:** 10.1155/2020/9731854

**Published:** 2020-04-27

**Authors:** Weihao Kong, Yuanyuan He, Huarui Bao, Wenlong Zhang, Xingyu Wang

**Affiliations:** ^1^Department of Emergency Surgery, Department of Emergency Medicine, The First Affiliated Hospital of Anhui Medical University, Heifei, China; ^2^Department of Emergency Internal Medicine, Department of Emergency Medicine, The First Affiliated Hospital of Anhui Medical University, Heifei, China

## Abstract

**Background:**

Acute pancreatitis (AP) is a life-threatening disease caused by a variety of factors, and once it progresses to severe acute pancreatitis, the prognosis is poor. The purpose of this study was to investigate the diagnostic value of the neutrophil-lymphocyte ratio (NLR) for predicting the severity of acute pancreatitis.

**Materials and Methods:**

We searched the databases of PubMed, EMBASE, Web of Science, and Cochrane Library to identify eligible studies using the NLR to predict the severity of AP. The sensitivity (SEN), specificity (SPE), negative likelihood ratio (NLR), positive likelihood ratio (PLR), diagnostic odds ratio (DOR), and area under the receiver operating characteristic curve (AUC) were combined using a bivariate mixed model.

**Results:**

A total of 10 articles containing 394 cases and 1319 controls were included in the study. The combined SEN, SPE, NLR, PLR, DOR, and AUC are 79% (73%-84%), 71% (59%-80%), 0.30 (0.21-0.41), 2.7 (1.8-4.0), 9 (5-18), and 0.82 (0.78-0.85), respectively.

**Conclusions:**

NLR has a moderately high diagnostic value in predicting the severity of acute pancreatitis.

## 1. Introduction

Acute pancreatitis (AP) is a disease characterized by dysfunction of pancreatic acinar cells, improper activation of trypsin, and subsequent destruction of pancreatic self-defense mechanisms, further exacerbating injury and damage of pancreatic cells [1]. It is a rapidly developing inflammatory process of the pancreas, and the most common reasons are alcohol and gallstones [2, 3]. As one of the most common gastrointestinal diseases in hospitalized patients, the incidence of AP has gradually increased and is 4.9 to 73.4 cases per 100,000 people worldwide in the past few decades, imposing a heavy burden on the health system and leading to long-term hospitalization, most medical costs, and significant mortality [1, 2, 4].

10% to 20% of AP patients will develop SAP, and the leading cause of poor prognosis in patients with AP is a vital organ (cardiovascular organs, lung, and kidney) failure and pancreatic necrosis [5, 6]. In clinical practice, varieties of scoring systems are available and have been gradually confirmed, such as the Ranson score, Glasgow score, Acute Physiology and Chronic Health Evaluation (APACHE II), BISAP, and computed tomography severity index (CTSI) [6]. These systems are cumbersome and take a long time to operate, requiring a lot of parameters that are not routinely collected in the early stages of the disease [4, 7]. For example, the BISAP score is characterized by high specificity, but its sensitivity to SAP is not satisfied [8]. Therefore, their early prediction power is not good.

In AP, inflammation first activates a series of inflammatory cytokines, proteolytic enzymes, and anaerobic radioactive nucleic acids to destroy the tissue [9]. The degree of neutrophils decrease is related to the improvement of prognosis of AP, while the degree of lymphocyte increase is related to the severity of the disease [9]. The neutrophil-lymphocyte ratio (NLR) is a more comprehensive biomarker that used neutrophil and lymphocyte counts to respond rapidly to the extent of inflammatory progression and serves as a useful predictive marker to identify the severity of AP. It is well known that AP is a fast-onset inflammation of the pancreas, and an effective prediction of the severity of AP can guide AP patients to receive adequate treatment earlier, contributing to a better prognosis [9]. NLR could be used as an independent predictor of the severity of pancreatitis. However, the predictive value of NLR reported in the literature is inconsistent. Hence, this meta-analysis was carried out to study the significance of NLR in predicting the severity of AP.

## 2. Materials and Methods

### 2.1. Search Strategy of the Study

The literature on neutrophil-lymphocyte ratio and pancreatitis before September 2019 was searched in the database, including PubMed, EMBASE, Web of Science, and Cochrane Library, using the following keywords: “neutrophil to lymphocyte ratio” or “NLR” and “pancreatitis.” At the same time, manual retrieval was conducted independently by three members (Kong WH, He YY, and Bao HR) on the list of literature included in the study to ensure the quality of the research.

### 2.2. Inclusion and Exclusion Criteria

The inclusion criteria of this study are as follows: (1) all participants were diagnosed with AP; (2) the clinical predictive value of the NLR in patients with pancreatitis was evaluated in the article; (3) sufficient and completed data are available for calculating predictive indicators, including sensitivity, specificity, true positive rate (TP), false positive rate (FP), false negative rate (FN), and true negative rate (TN); and (4) studies are being published in English or Chinese.

The exclusion criteria of this study are as follows: (1) case reports, reviews, basic research, animal experiments, and conference abstracts; (2) studies lacking adequate data; and (3) articles that appear to be duplicated.

### 2.3. Data Extraction and Quality Assessment

Two group members (Kong WH and He YY) independently extracted data from the included studies, and disagreement was resolved by consulting a third group member through group discussion. For each study included, the following information was extracted: first author, country, publication year, true positive, false positive, false negative, true negative, cases, controls, etiology, sample collecting time, area under the receiver operating characteristic curve, cut-off value, sensitivity, and specificity. Based on the 2012 Atlanta classification criteria, we classified patients into mild acute pancreatitis (MAP), moderately severe acute pancreatitis (MASP), and severe acute pancreatitis (SAP). MAP refers to no organ dysfunction and local complications; MSAP refers to the occurrence of transient organ dysfunction (≤48 h) or accompanied by local or systemic complications; SAP refers to the existence of persistent organ dysfunction (>48 h) [10]. The quality of all included studies was systematically assessed using the Quality Assessment of Diagnostic Accuracy Study-2 (QUDAS-2) guidelines.

### 2.4. Statistical Analysis

All statistical analyses were performed using STATA 14.0 (Stata, College Station, TX, USA). The bivariate random effects model was used to merge the effect sizes of the included studies. The Spearman correlation analysis was used to explore whether threshold effects existed between studies and nonthreshold effects were evaluated by chi-square analysis and *I*^2^ statistics (*I*^2^ > 50% or *p* < 0.05 indicated significant heterogeneity among the included studies). Subgroup analysis and meta-regression were used to explore the sources of heterogeneity further. Moreover, the Fagan diagram is used to explore the relationship between the pretest probability, likelihood ratio, and the posttest probability. If the AUC is close to 1, the diagnostic power is good. Publication bias was assessed using Deeks' plot. A *p* value of less than 0.05 was considered statistically significant.

## 3. Results

### 3.1. Literature Search and Study Characteristics

**Figure 1:**
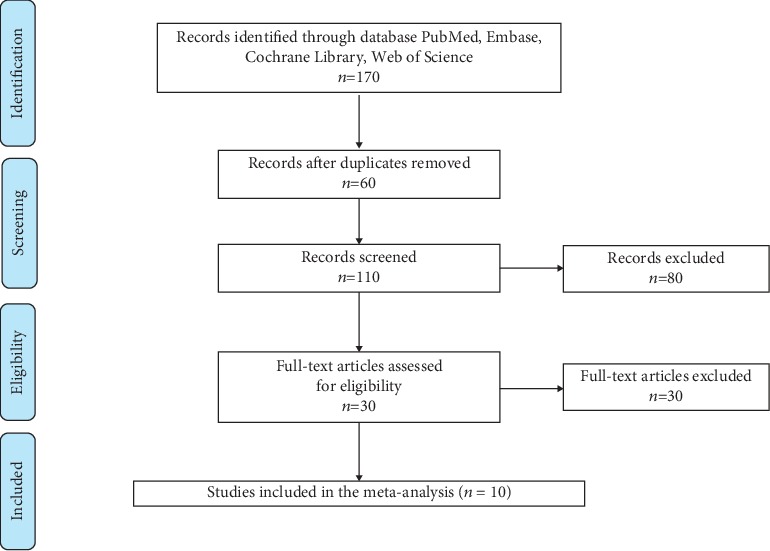
Flow chart of the study selection.

By searching PubMed, EMBASE, Web of Science, and Cochrane Library strictly in accordance with the inclusion and exclusion criteria, we enrolled a total of 10 eligible studies with an overall sample size ranging from 89 to 490 cases in countries including China, South Korea, the United States, Turkey, and the United Kingdom.  Figure 1 shows the detailed literature screening process.

Table 1 shows the essential characteristics of the studies included in this meta-analysis, including the author, country, publication year, TP, FP, FN, TN, case/control, etiology, collecting time, AUC, cut-off, sensitivity, and specificity. The sample collection time of the included study was at the time of admission, one day after admission, and within 3 hours after admission. The fluctuation of the area under the ROC curve ranges from 0.620 to 0.906. The summary of the included research is displayed in Table 2 with the subgroup of the population, sample, and publication year. Seven studies were on Asian populations, and seven out of 10 studies had a sample size of more than 100. Quality assessment is based on the Quality Assessment of Diagnostic Accuracy Study-2 (QUADAS-2). The QUADAS-2 scores for all the included studies were ≥4, indicating a moderately high quality for the included studies (Supplementary file 1).

### 3.2. Data Analysis

**Figure 2:**
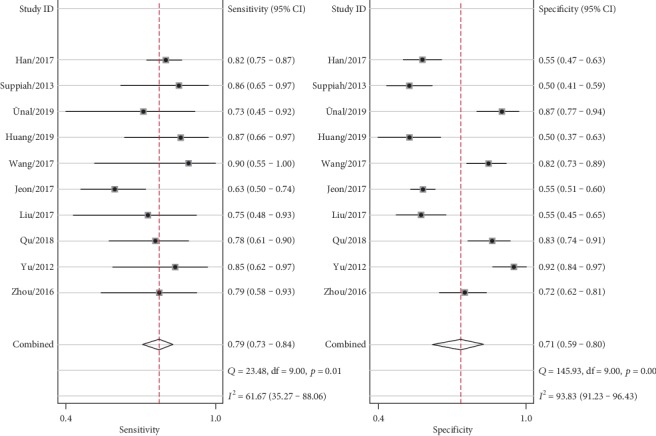
Forest plot of combined sensitivity and specificity.

**Figure 3:**
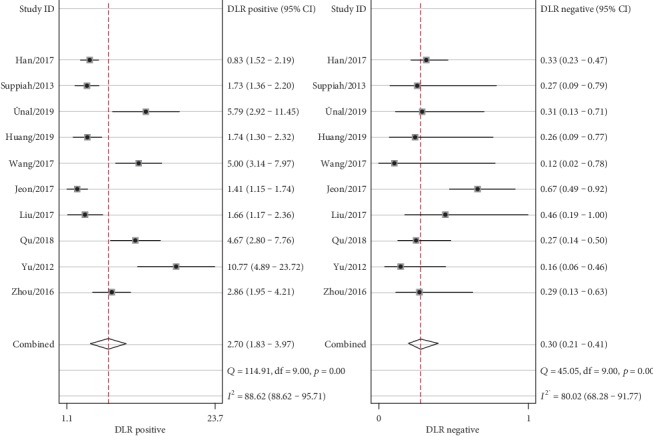
Forest plot of combined positive and negative likelihood ratios.

**Figure 4:**
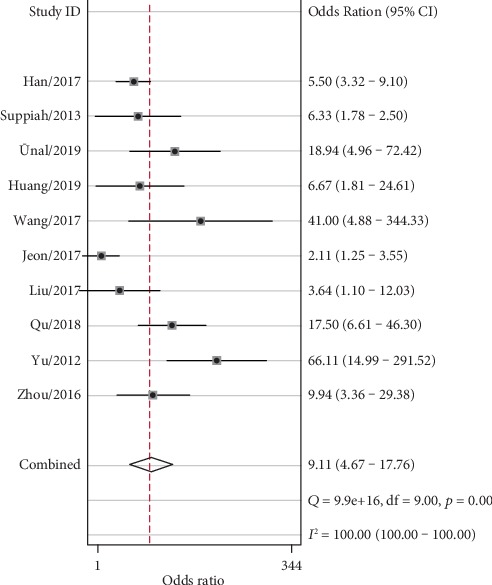
Forest plot of the combined diagnostic odds ratio.

**Figure 5:**
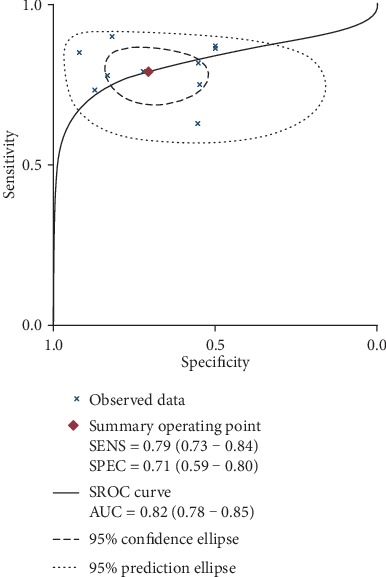
Combined AUC in all included studies.

**Figure 6:**
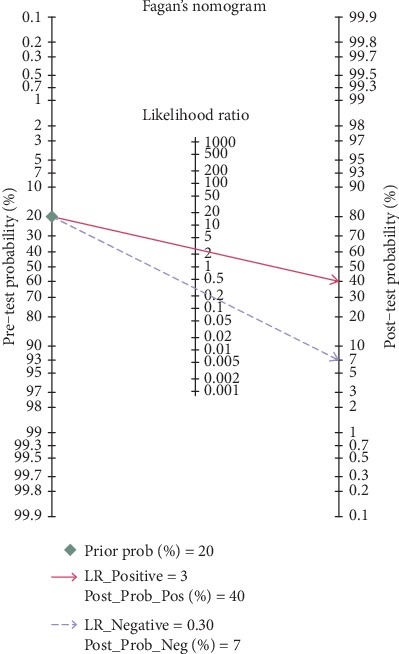
Fagan plot for evaluating the posttest probabilities.

The combined effects of sensitivity, specificity, positive likelihood ratio, negative likelihood ratio, diagnostic odds ratio, and area under the ROC curve were 79% (73%-84%), 71% (59%-80%), 2.7 (1.8-4.0), 0.30 (0.21-0.41), 9 (5-18), and 0.82 (0.78-0.85), respectively. (Figures 2–5). The AUC was 0.82, which shows that it had a moderately high predictive value. The results of Figure 6 show that when the neutrophil-lymphocyte ratio predicts the severity of acute pancreatitis by 20%, a positive result increases the posttest probability of severe pancreatitis to 40%, while a negative result reduces the posttest probability of severe pancreatitis to 7%. All results suggested that NLR has a moderately high value in predicting the severity of AP patients.

#### 3.2.1. Threshold Effects and Heterogeneity

Significant heterogeneity between studies cannot be avoided, despite the strict inclusion and exclusion criteria. It is well known that the main causes of heterogeneity are the threshold effect, nonthreshold effect, and publication bias. The threshold effect between studies can be determined by calculating the Spearman correlation coefficient between sensitivity and specificity for all included studies. The results of the Spearman correlation analysis show that the correlation coefficient is -0.109 (*p* value is 0.763), so there is no threshold effect. The *I*^2^ of sensitivity and specificity was 61.67 and 93.83, respectively, indicating significant heterogeneity. Therefore, further subgroup analysis and meta-regression were needed to explore the sources of heterogeneity of sensitivity and specificity.

#### 3.2.2. Metaregression and Robustness Tests

To further explore the sources of heterogeneity, we performed subgroup analysis and metaregression for the following variables: sample size, country, and publication year. The results of the subgroup analysis showed that there was no significant difference in the subgroup of the Asian population, sample size greater than 100, and publication year later than 2015. Then, we conducted metaregression, and the results showed that no statistically significant variables were detected (Supplementary file 2). Finally, a sensitivity analysis was carried out, and the results of goodness of fit and bivariate normality analyses (Supplementary file 3(a) and 3(b)) showed that the bivariate model was moderately robust. We also performed influence analyses and further excluded one outliner (Study 5). After exclusion, the AUC has not changed, the NLR dropped from 0.30 to 0.26, and the sensitivity, specificity, PLR, and DOR have risen from 79% to 81%, 71% to 72%, 2.7 to 2.9, and 9 to 11, respectively, which shows no significant change after excluding the outliner.

#### 3.2.3. Publication Bias

**Figure 7:**
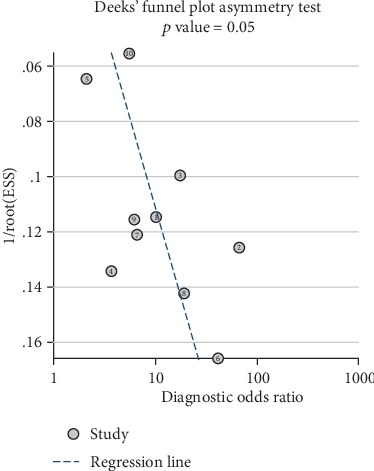
Detection of publication bias.

Deeks' funnel plot asymmetry test assessed publication bias in this pooled analysis, which indicated obvious publication bias (*p* = 0.05) (Figure 7).

## 4. Discussion

AP is a potentially life-threatening disease caused by a variety of factors, including gallstones, alcohol abuse, hypercalcemia, drug poisoning, and gene mutation [11]. The underlying pathophysiology in which local pancreatic injury drives the systemic inflammatory response has not been fully elucidated, but cumulative data suggest that both the innate immune system (including neutrophils, monocytes, and macrophages) and adaptive immune system (mainly composed of lymphocytes) play pivotal roles in the progression of the disease [12, 13]. According to the revised Atlanta classification standard, AP can be classified into mild, moderate, or severe [14]. The overall mortality rate for AP is about 5%. If AP is not treated promptly, the prognosis is generally poor.

In recent years, the APACHE-II scoring system and Ranson score have been of great significance in assessing the severity of disease in patients with acute pancreatitis. However, the APACHE-II scoring system needs to collect many indicators, and some indicators cannot be collected in ordinary wards. The Ranson score takes 48 hours to complete, which will miss the best time for treatment [15]. As a common indicator of routine blood tests, NLR has the advantages of rapid detection, high sensitivity, inexpensiveness, and non-invasiveness and has gradually attracted the attention of many researchers. Based on the systematic review and meta-analysis of Gao et al., we found that the SEN, SPE, and AUC of the APACHE-II scoring system for predicting SAP were 83%, 59%, and 0.82, respectively. The SEN, SPE, and AUC of the Ranson score for predicting SAP were 66%, 78%, and 0.83, respectively [8]. In this study, we found that the combined SEN, SPE, and AUC are 79%, 71%, and 0.82, respectively. When compared with the APACHE-II scoring system and the Ranson score, the NLR has moderate sensitivity and specificity and the same diagnostic value. Hence, the neutrophil-lymphocyte ratio may be a potential marker that can be used to distinguish patients with severe pancreatitis and mild to moderate pancreatitis patients. This provides an essential strategy for the treatment and supervision of patients with acute pancreatitis.

The results of the heterogeneity test found significant heterogeneity among these studies, so we performed subgroup analysis, metaregression, robustness testing, and publication bias detection. The results of the subgroup analysis showed that there was no significant difference between the combined effect size and the overall effect size in different subgroups, while the metaregression did not detect significant factors affecting heterogeneity. Sensitivity analysis and outlier detection found that Study 5 may be a potential outlier, so we combined the effect sizes of the studies after excluding the outlier and found that there is no significant influence on the overall effect, so our results are stable and reliable. The detection of publication bias found potential publication bias, suggesting that this may be a significant source of heterogeneity.

The excessive recruitment of neutrophils, the center to the progression of SAP inflammation, will cause a large number of cells to migrate into the inflammatory site and lead to a sustained inflammatory response and local tissue damage that mediates remote multiorgan injury or subsequent death [12, 16, 17]. Acute systemic inflammatory responses have been associated with changes in neutrophilia and lymphocytopenia, which reflects dramatic changes in the immune system in the AP environment [12]. And NLR represents a balance between inflammatory activator neutrophils and inflammatory regulator lymphocytes, and the higher the value, the more unbalanced the inflammatory state [18, 19]. Among many inflammatory markers, an elevated NLR is a reliable and sensitive inflammatory marker that can be a valuable predictor of poor outcomes in patients with infection, sepsis, stroke, cardiovascular disease, and cancer [20].

Since Zahorec first reported that NLR is an easily measurable parameter of systemic inflammation, its prognostic value has been widely used in a variety of clinical conditions, such as inflammation, cardiovascular disease, and tumors [21]. At the same time, a growing number of studies have shown that NLR is associated with AP and is better than other serum markers in predicting the severity and prognosis of AP [9]. Huang et al. demonstrated that NLR is more sensitive and accurate than WBC in determining the severity of HTG-AP [18]. Abayli and Gencdal conducted a correlation study between the NLR and Ranson score on 435 AP patients and found that NLR is a simpler, more practical, and effective parameter for diagnosing the severity of AP compared with the current scoring system [22]. Li et al. showed that compared with PNI, CRP, RDW, and LMR, the NLR is the most powerful indicator of overall survival and has the highest prognostic value in AP patients [9]. O'connell et al. found that the NLR greater than 5 increases the risk of admission to ICU [23]. Suppiah et al. revealed that the NLR measured during the first 48 hours of hospitalization was significantly associated with the risk of developing a severe form of AP [24].

NLR has been shown to be associated with disease severity and adverse outcomes in AP [24, 25]. However, the potential association between NLR and prognosis in AP patients has not been assessed since the publication of the revised Atlanta classification. New evidence suggests that therapeutic interventions targeting neutrophils can significantly reduce tissue damage and prevent the progression of pancreatitis [16]. Since the prognosis of AP depends on its severity, identifying patients most likely to develop SAP after admission has important clinical implications that will help to classify and initiate aggressive early treatment [10].

The main advantage of our study is that it is the first meta-analysis to analyze the predictive value of NLR for the severity of AP. What is more, our analysis indicates that the NLR value has a moderately high predictive value, which can be used as a useful clinical tool for clinical guidance.

Indeed, the present meta-analysis has its shortcomings that should be considered. First, our study did not determine an optimal cut-off value for predicting the severity of pancreatitis. Second, the heterogeneity of our research is significant. Although the heterogeneity of our study was considerable, we performed subgroup analysis, meta-regression, and outlier detection in turn, and the results showed that the outcome was stable. Third, the studies we have included have a high proportion of data in the Asian population, which may lead to inevitable publication bias. Fourth, the NLR tends to change over time, and the dynamic changes of NLR may affect the reliability of the outcome. Fifth, considering comparative studies are lacking, and hence, the analysis is limited by the value of references. Finally, we only include articles written in Chinese or English, and we may miss relevant studies published in journals in other languages.

Early recognition and prediction are of great significance for the treatment of patients with SAP. The NLR has the advantages of being easily accessible and low cost and having moderately high diagnostic power in predicting the severity of AP patients, which provided useful clinical guidance for us to evaluate the severity of AP better.

## Figures and Tables

**Table 1:** General features of the included studies.

Author	Country	Year	TP	FP	FN	TN	Sample	Case/control	Etiology	Collecting time	AUC	Cut-off	Sensitivity (%)	Specificity (%)
Zhou	China	2016	19	26	5	68	118	24/94	Biliary/hyperlipidemia/alcoholic/other	1 day after admission	0.794	9.87	79.3	72.3
Yu	China	2012	17	6	3	70	96	20/76	NA	1 day after admission	0.906	7.85	85	92.11
Qu	China	2018	28	14	8	70	120	36/84	Biliary/hyperlipidemia/alcoholic/other	1 day after admission	0.794	9.863	76.9	83.8
Liu	China	2017	12	47	4	57	120	16/104	Biliary/nonbiliary	1 day after admission	0.653	11.93	75	54.8
Jeon	Korea	2017	44	187	26	233	490	70/420	Biliary/hyperlipidemia/alcoholic/idiopathic/cancer	1 day after admission	0.620	5.18	62.9	55.5
Wang	USA	2017	9	18	1	82	110	10/100	Hyperlipidemia	On admission	0.891	10	90	82
Huang	China	2019	20	33	3	33	89	23/66	Hyperlipidemia	1 day after admission	0.706	5.88	87	50
Ünal	Turkey	2019	11	9	4	62	96	15/81	Biliary/hyperlipidemia/alcoholic/ERCP	Within 3 hours after admission	0.747	7.94	73.3	76.5
Suppiah	UK	2013	19	62	3	62	146	22/124	Biliary/alcoholic/other	1 day after admission	0.764	8.1	86.4	50
Han	China	2017	129	76	29	94	328	158/170	Biliary/smoking/alcoholic/prior acute pancreatitis/other	1 day after admission	0.723	9.64	81.65	55.29

TP: true positive rate; FP: false positive rate; FN: false negative rate; TN: true negative rate; NA: not available.

**Table 2:** Subgroup analysis stratified by population, sample, and publication year.

Category	No. of studies	SEN (95% CI)	SPE (95% CI)	NLR (95% CI)	PLR (95% CI)	DOR (95% CI)	AUC (95% CI)
Population							
Asian	7	78% (71%-84%)	68% (54%-80%)	0.32 (0.21-0.47)	2.5 (1.6-3.9)	8 (4-17)	0.81 (0.77-0.84)
Sample							
>100	7	78% (70%-84%)	66% (55%-75%)	0.33 (0.23-0.49)	2.3 (1.6-3.2)	7 (4-13)	0.80 (0.76-0.83)
Publication year							
>2015	8	78% (70%-84%)	0.69 (0.58-0.79)	0.32 (0.23-0.46)	2.5 (1.8-3.6)	8 (4-15)	0.81 (0.77-0.84)
Overall	10	79% (73%-84%)	0.71 (0.59-0.80)	0.30 (0.21-0.41)	2.7 (1.8-4.0)	9 (5-18)	0.82 (0.78-0.85)
Outliers excluded	9	81% (76%-85%)	0.72 (0.60-0.82)	0.26 (0.20-0.34)	2.9 (2.0-4.4)	11 (6-21)	0.82 (0.79-0.85)

SEN: sensitivity; SPE: specificity; NLR: negative likelihood ratio; PLR: positive likelihood ratio; DOR: diagnostic odds ratio; AUC: area under the receiver operating characteristic curve.

## Data Availability

The data used to support the findings of this research are available from the corresponding author upon request.

## Supplementary Materials

Supplementary 1 Quality assessment of included studies.

Supplementary 2 Metaregression based on population, sample, and publication year. Explore potential factors that caused heterogeneity based on sample size, population, and publication year. The results of metaregression show that none of these variables is a potential source of heterogeneity.

Supplementary 3 Sensitivity analysis of included studies. The results of goodness of fit and bivariate normality show that the bivariate model has moderate stability (a and b). The results of sensitivity analysis and outlier detection identified that Study 5 was an outlier (c and d).
