# Supplementary figures and images for: Diagnostic Value of Neutrophil-Lymphocyte Ratio for Predicting the Severity of Acute Pancreatitis: A Meta-Analysis
Source: Dis Markers. 2020 Apr 27;2020:9731854. doi: 10.1155/2020/9731854 (PMC7232731; doi:10.1155/2020/9731854)

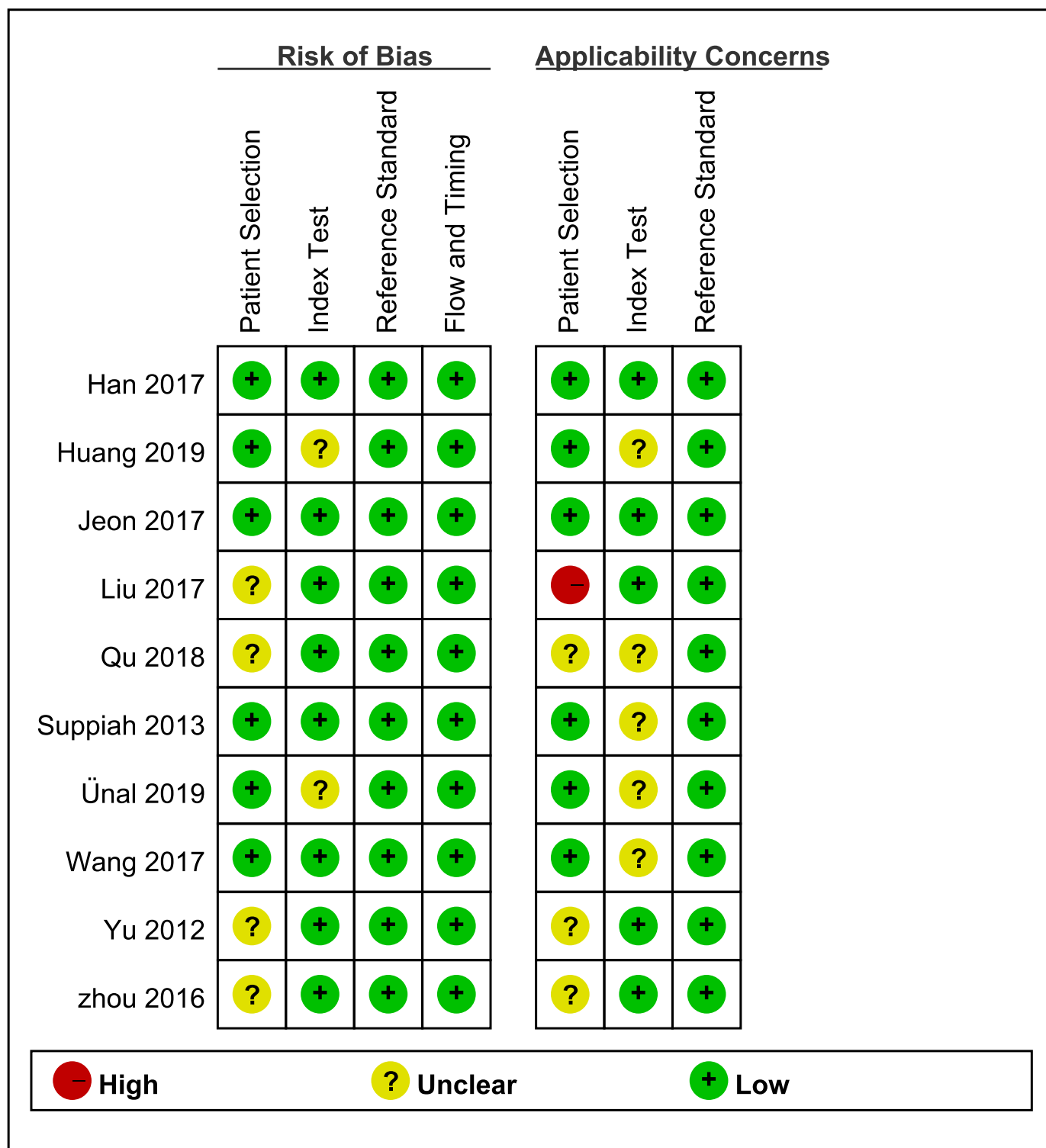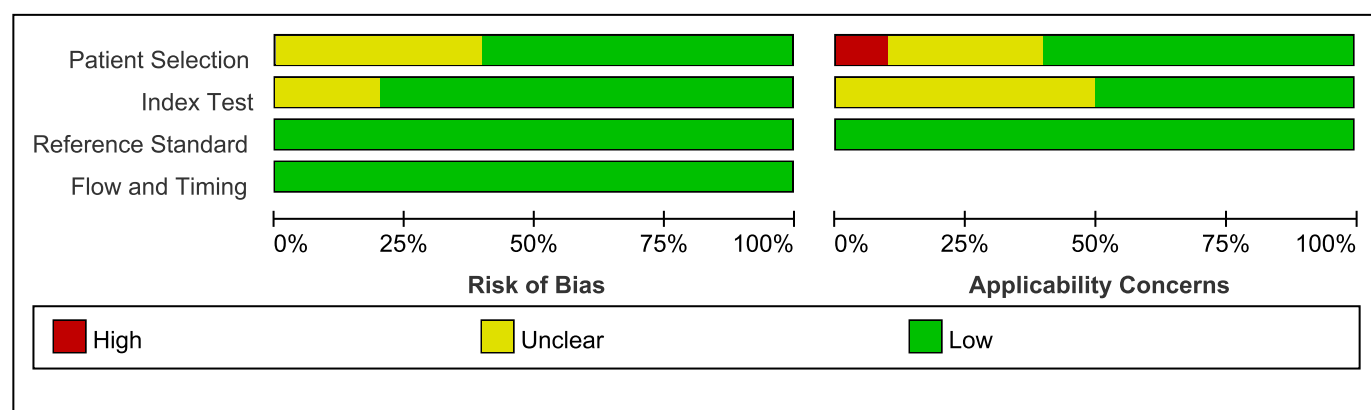

Supplement: Supplementary 1 — Quality assessment of included studies. [file 9731854.f1.pdf]

## Univariable Meta-regression & Subgroup Analyses

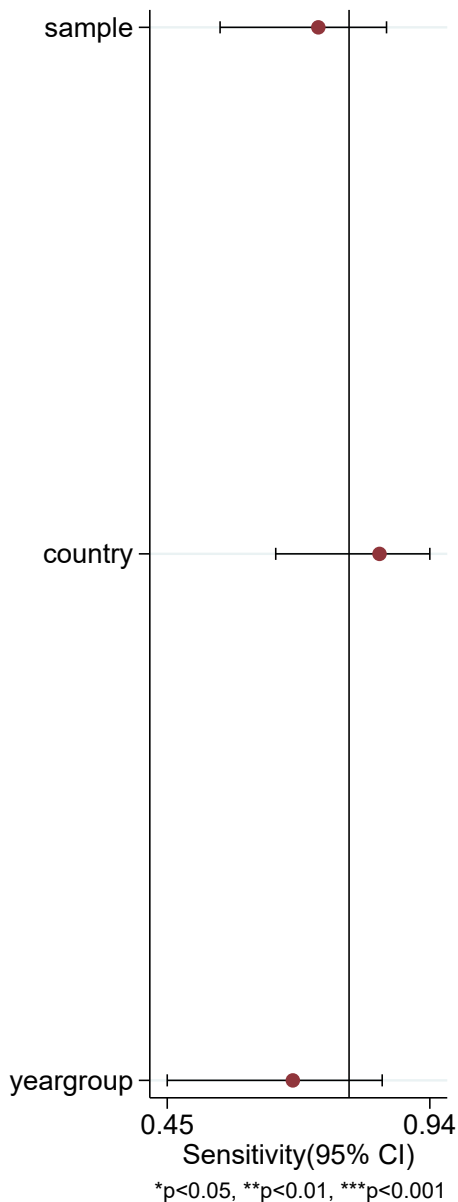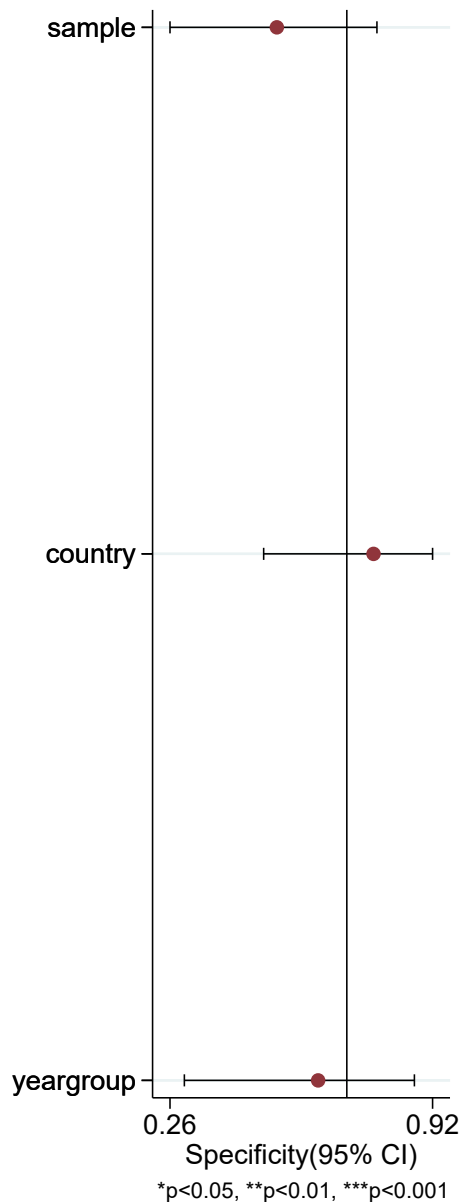

Supplement: Supplementary 2 — Metaregression based on population, sample, and publication year. Explore potential factors that caused heterogeneity based on sample size, population, and publication year. The results of metaregression show that none of these variables is a potential source of heterogeneity. [file 9731854.f2.pdf]

(a) Goodness-Of-Fit

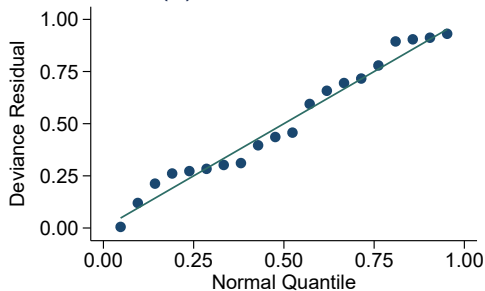

(b) Bivariate Normality

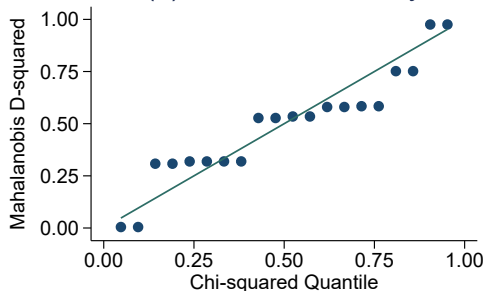

(c) Influence Analysis

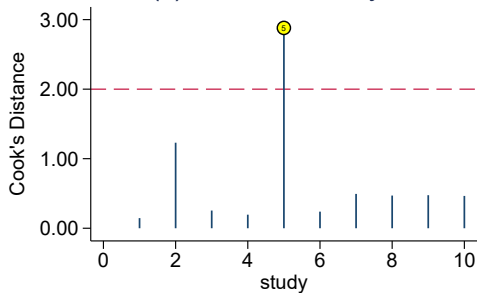

(d) Outlier Detection

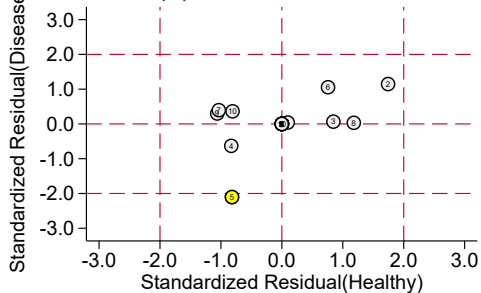

Supplement: Supplementary 3 — Sensitivity analysis of included studies. The results of goodness of fit and bivariate normality show that the bivariate model has moderate stability (a and b). The results of sensitivity analysis and outlier detection identified that Study 5 was an outlier (c and d). [file 9731854.f3.pdf]
